# The turnover challenge in centers for disease control and prevention: role of salaries, job satisfaction and burnout—a cross-sectional study in a province of China

**DOI:** 10.3389/fpubh.2026.1850669

**Published:** 2026-06-05

**Authors:** Hanlin Nie, Wanjin Yang, Jingting Zeng, Qian Bai, Elizabeth Maitland, Stephen Nicholas, Yijie Wang, Xuefeng Shi

**Affiliations:** 1 School of Management, Beijing University of Chinese Medicine, Beijing, China; 2 Office of Labor and Social Security, School of Management, Tianjin University of Traditional Chinese Medicine, Tianjin, China; 3 School of Management, University of Liverpool, Liverpool, England; 4 Health Services Research and Workforce Innovation Center, Newcastle Business School, University of Newcastle, Newcastle, NSW, Australia; 5 Australian National Institute of Management and Commerce, Sydney, NSW, Australia; 6 School of Public Health, Capital Medical University, Beijing, China; 7 National Institute of Traditional Chinese Medicine Strategy and Development, Beijing University of Chinese Medicine, Beijing, China

**Keywords:** burnout, job satisfaction, public health workforce, salary, turnover intention

## Abstract

**Introduction:**

As a core pillar in China's public health system, Centers for Disease Control and Prevention (CDCs) face significant workforce turnover. Using data from a certain province in China, our study addresses two gaps: the structural relationships and the combined effect on CDC turnover of salary, job satisfaction, and burnout, and, second, the inter-relationship of marital status and these factors.

**Methods:**

From March to April 2024, a cross-sectional survey was conducted in the study province of China among CDC staff, collecting data on salary, job satisfaction, burnout, turnover intention, and sociodemographic information. Chained mediation models analyzed the multiple mediating effects of job satisfaction and burnout between salary and turnover intention.

**Results:**

Lower salary was directly associated with higher turnover intention (β = −0.059, *P* < 0.05), accounting for 42.14% of the total effect. Job satisfaction and burnout demonstrated significant chained mediation between salary and turnover intention. Job satisfaction also exerted an independent mediating effect (β = −0.058, *P* < 0.01), while burnout did not. The total indirect effect coefficient was −0.081, constituting 57.86% of the total effect. The indirect chain-link effect pathways for married staff were similar to the full-sample pathways, though effect sizes differed. Unmarried staff reported significantly higher burnout than married staff (6.94 vs. 6.31, *P* < 0.01), but showed no significant associations between salary and job satisfaction, burnout, or turnover intention.

**Discussion:**

Job satisfaction and burnout served as chained mediators between salaries and turnover intentions, with job satisfaction exerting an additional independent mediating effect. Internal heterogeneity was revealed within the marriage subgroups. Interventions should prioritize reviewing and improving the compensation system, enhancing job satisfaction, reducing burnout, and implementing differentiated measures to reduce workforce turnover.

## 1 Background

Centers for Disease Control and Prevention (CDCs) are a core pillar in China's public health system (1), responsible for disease prevention and control, public health emergency responses, vaccination monitoring, and public health promotion (1, 2). Since 2009, the continuous reforms in China's health sector have seen CDCs make significant improvements both in their institutional scale and operational capabilities (2, 3), including increasing the number of CDC staff to 250,000 by 2025 (4). Due to the sudden and contagious nature of disease prevention and control work, CDC staff frequently face significant on-the-job challenges, including heavy workloads, uncertainties in infectious disease risks, and emotional stress (5). Numerous studies confirm that CDC employees receive compensation disproportionately lower than their counterparts in medical institutions at equivalent levels (6, 7). China's CDC is experiencing severe staff turnover, particularly among highly educated backbone staff and young professionals (8–11), which is especially pronounced in China's central and western provinces (3, 12). Research on the attrition of CDC personnel generally agrees that low salary is the key factor constraining recruitment and explaining high turnover (9). Studies also indicate the rise in mental health issues, with low job satisfaction and burnout serving as significant accelerators of staff turnover (5, 13, 14).

Across diverse countries and professions, empirical research demonstrates that declining job satisfaction is associated with burnout, and higher levels of burnout are correlated with stronger intentions to leave jobs, which in turn are associated with higher turnover rates (15–17). Within the context of China's health workforce, the job satisfaction-burnout-turnover intention relationship has mainly been studied among urban doctors and nurses (18, 19) and rural primary health care providers (20), with fewer studies of public health professionals, particularly the largest group, CDC staff. Some studies on CDC staff have confirmed that low salary is associated with reduced job satisfaction or increased burnout, or a higher likelihood of resignation (7, 11, 21). These studies have largely been limited to analyzing the binary relationships between job satisfaction and burnout, or burnout and turnover intention, or job satisfaction and salary.

The structural relationships and combined effect among these variables, especially on turnover, have not been fully analyzed for CDC personnel. In addition, the well-established impact of marital status on employees' turnover behavior (22) has been neglected in CDC turnover studies. Non-CDC studies indicate that married employees may be more likely to remain in an organization due to family responsibilities or the need for stable work than unmarried staff (14, 23). A stable marital life can provide emotional support and alleviate work-related stress, reducing burnout and lowering turnover rates (24–26). Differences in the impact of marital status on job satisfaction, burnout, and turnover intentions among CDC staff remain unexplored. Our study addresses two gaps in the existing literature: the structural relationships and combined effect on CDC turnover of salary, job satisfaction, and burnout, and the inter-relationship of marital status and turnover, salaries, job satisfaction, and burnout. Understanding these relationships is critical to developing targeted intervention measures to improve salaries and job satisfaction, alleviate burnout, and reduce turnover intentions among CDC staff. China's CDC system faces exceptionally high workloads and pressure due to China's complex terrain and climate, a vast population, diverse disease profiles, regional economic disparities, and linguistic-cultural heterogeneity (27–29). We conducted a cross-sectional survey of all CDCs in a selected province in China to explore the mediating role of job satisfaction and burnout between salary and turnover intention. Additionally, we conduct a subgroup analysis based on marital status to explore its heterogeneity.

The empirical evidence suggests that salary significantly impacts job satisfaction, burnout, and turnover intention (7, 11, 30). The setting of salary levels should align with the value of labor, fully meet employees' living and development needs, and remain consistent with social equity (11, 31). Before 2017, the CDC budget provided fee-based services, such as health screenings, preventive physical examinations, and epidemic prevention, with a portion of the additional revenue subsidizing employee salaries (32). When the Chinese government eliminated the CDC health service fees in 2017, falling CDC income reduced the CDC budget and squeezed employee salaries (32). Subsequent government fiscal support failed to fully compensate for this income shortfall, leading to job dissatisfaction and resignations among staff due to reduced salaries (33). While current research generally agrees that CDC personnel salaries require improvement (7, 9, 21), our study explores the complex relationship between salaries, job satisfaction, reduced burnout, and turnover rates among CDC staff.

Job satisfaction refers to the degree to which employees are satisfied with their job roles, work environment, and organizational policies, and it is a primary factor influencing employees' work motivation and initiative (34). Job satisfaction among Chinese CDC personnel is relatively low, as studies from different regions and different CDC branches have consistently concluded (35–37). Since job satisfaction is a multidimensional concept, the causes of low satisfaction are complex and have been primarily attributed to factors such as low salary, unclear career advancement prospects, high work pressure, and low job recognition (5, 35). Low job satisfaction is also associated with derivative issues, including low work efficiency, frequent absenteeism, burnout, and high employee turnover (38).

Burnout is a psychological syndrome resulting from prolonged exposure to emotional and interpersonal stressors in the workplace (39). Burnout comprises three dimensions: emotional exhaustion, depersonalization, and diminished personal accomplishment (40). High levels of burnout are common among Chinese CDC staff (7). Burnout among CDC personnel has been linked to work intensity, compensation and benefits, job satisfaction, family responsibilities, and responses to public health emergencies (41). High burnout is associated with numerous health and psychological problems, such as emotional exhaustion, low sense of achievement, headaches and insomnia, workforce instability, and impaired work efficiency, including lack of concentration and frequent errors (7).

Turnover intention has been identified as a critical factor constraining the development of China's CDC (11). Turnover intention is typically defined as an employee's psychological tendency to leave their current organization for other jobs (42). A complex decision-making process, job resignation can be broadly divided into three main interacting categories: internal factors, such as work pressure, fairness sense, and promotion expectations (11, 43); external factors, for example, salary, employment opportunities, and family responsibilities (7); and psychological factors, including job satisfaction, burnout, and professional identity (5). Reducing turnover intentions among CDC personnel has become a significant challenge.

**Figure 1:**
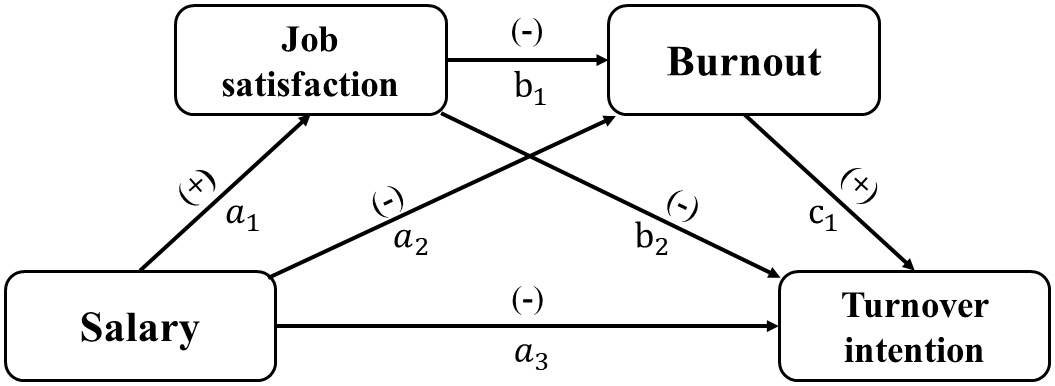
Model of the hypothesized mediating role.

Among healthcare workers (18, 20), increasing salary can enhance job satisfaction (44), reduce the occurrence of burnout (45), and decrease turnover intention (46). Improved job satisfaction can lower turnover intention by reducing burnout. As shown in Figure 1, our study constructs a chain-mediated model of the salary-job satisfaction-burnout-turnover intention relationship, and proposes the following hypotheses:

❖ Hypothesis 1: Salary is negatively associated with turnover intention among CDC staff.
❖ Hypothesis 2: Among CDC staff, job satisfaction mediates the relationship between salary and turnover intention.
❖ Hypothesis 3: Among CDC staff, burnout mediates the relationship between salary and turnover intention.
❖ Hypothesis 4: Among CDC staff, job satisfaction and burnout jointly mediate the relationship between salary and turnover intention in a chain-mediated manner.

## 2 Methods

### 2.1 Participants

Using whole-cluster sampling, we conducted a cross-sectional survey from March to April 2024 at all CDCs in the study province. Due to the gradual reduction in the number of staff allocated to each provincial, prefectural, and county level CDC, 80 provincial level, 50 prefecture level, and 30 county level CDC staff were randomly selected from each CDC. The quota was implemented by randomly selecting individuals from the rosters of on-duty staff at each CDC during the survey period, using a computer-generated random number after assigning codes to the names. A total of 7,929 respondents completed the survey, with 6,343 respondents passing the strict consistency test, with an effective participation rate of 80%. Our study relied on a web-based survey platform (wjx.com) to distribute the electronic anonymous questionnaire and set up strict quality control. The wording and layout of the questionnaire were adjusted through two rounds of discussions with experts and the project team. The questionnaire included one consistency check question and three logic check questions, with responses not meeting the checks excluded. At each CDC branch, a trained data enumerator assisted in completing data collection and providing online and telephone consultations. All participants responded anonymously and gave informed consent. The study was approved by the Ethics Committee of Beijing University of Traditional Chinese Medicine (NO.2024BZYLL0303).

### 2.2 Measures

#### 2.2.1 Socio-demographic characteristics

We collected data on gender, age, *bianzhi* (tenured employment position in China) (47), education, title, whether they had worked overtime in the last six months, number of chronic diseases they had, marital status, and CDC level. Marital status was categorized as married and unmarried (including single and divorced).

#### 2.2.2 Salary

In this study, salary refers to the average monthly sum of the basic wage plus bonuses, allowances, overtime pay, and any additional remuneration. The scope of salary was clearly defined in the questionnaire, and specific data were obtained through respondents' reports of their average monthly pre-tax income over the past year. The processing of salary data involved four steps. First, sample entries with missing salary data or abnormal entries (such as non-numeric values) were deleted. Second, logical consistency checks were performed by cross-referencing each salary report with the respondent's administrative level (province/prefecture/county), professional title, educational attainment, and employment status. Values exceeding 50% of the mean for the corresponding group were flagged and either adjusted (taking into account job responsibilities and working hours) or excluded, as appropriate. Third, a 5% winsorization method was applied to mitigate the influence of outliers. Values below the 2.5th percentile and above the 97.5th percentile were replaced with the corresponding percentile values. Fourth, the salary data was converted from Chinese Yuan (CNY) to United States Dollar (USD); for regression analysis, a natural logarithm transformation [ln(salary)] was applied to correct for positive skewness.

#### 2.2.3 Job satisfaction

Using a pairwise translation, the *Minnesota Satisfaction Questionnaire* (MSQ) was adopted (48). Two experts specialized in health management translated the MSQ into Chinese; English professionals back-translated the Chinese questionnaire into English; and any anomalies in the Chinese version were adjusted by the team. The final Chinese version of the MSQ was finalized after two rounds of validation and one pilot survey. A total of 20 items were included, measured by a 5-point Likert scale (1-very dissatisfied to 5-very satisfied). Job satisfaction scores were averaged over all items, with higher scores indicating higher job satisfaction. The MSQ has been widely applied in job satisfaction studies across numerous occupations in China, such as bank staff (49), civil aviation pilots (50), police officers (51), and anaesthesiologists (52), demonstrating its well-established validity. In the present research, the Cronbach's alpha coefficient for the reliability test was 0.926, and the Kaiser-Meyer-Olkin Measure (KMO) for the total validity test was 0.949 (*P* < 0.001). The Bartlett test of sphericity showed *P* < 0.001, and the fit indices of the confirmatory factor analysis (CFA) indicated that the scale had a Goodness-of-Fit Index (GFI) > 0.9 and Root Mean Square Error of Approximation (RMSEA) < 0.05, suggesting good construct validity.

#### 2.2.4 Burnout

The same procedures applied to the MSQ were used for the *Maslach Burnout Inventory–General Survey* (MBI-GS) (40, 53). The 15 items were divided into three sub-dimensions, including five emotional exhaustion, four depersonalization, and six diminished personal accomplishment items (40). Each item was measured by a 7-point Likert scale, with 0 “never” to 6 ‘every day'. The score for each sub-dimension was the average score of the items, with reverse scoring applied to the diminished personal accomplishment. The score for burnout was the sum of the 3 sub-dimensions. A higher score indicates more severe burnout. The threshold for detecting burnout was set at 25% of the total score; that is, a total score > 4.5 was considered a positive result for burnout (54). The MBI-GS has been widely used in China to assess staff burnout levels, such as among social welfare workers (55), nurses (56), and grassroots officials (57), demonstrating good validity. In the present research, the Cronbach's alpha was 0.831, and the KMO value was 0.904 (*P* < 0.001). The Bartlett test of sphericity showed *P* < 0.001, and the fit indices of the CFA indicated that the scale had a GFI > 0.9 and RMSEA < 0.05, suggesting good construct validity.

#### 2.2.5 Turnover intention

Following the MSQ and MBI-GS procedure, the four-item *Intent to Leave Scale* (ILS) was adopted (58). Each item was measured by a 5-point Likert scale (1- strongly disagree to 5- strongly agree), with the score averaged over 4 items. A higher score indicates a stronger turnover intention. The ILS has been applied in studies examining job turnover among kindergarten teachers (59) and nurses (60) in China, demonstrating good validity. In the present research, the Cronbach's alpha was 0.806, and the KMO value was 0.775 (*P* < 0.001). The Bartlett test of sphericity showed *P* < 0.001, and the fit indices of the CFA indicated that the scale had a GFI > 0.9 and RMSEA < 0.05, suggesting good construct validity.

### 2.3 Statistical analysis

Descriptive statistics, Chi-square test, and Kruskal-Wallis H test were used to analyze the scores of subgroup differences in job satisfaction, burnout, and turnover intention for different demographic characteristics. Harman's one-way statistical control was used to check the effect of common method bias (61, 62). Pearson correlation coefficients were used to examine bivariate associations among salary, job satisfaction, burnout, and turnover intention. We applied Cohen's (1988) effect size criteria for correlations (|r| ≥ 0.1 small, ≥ 0.3 medium, ≥ 0.5 large). Owing to the large sample size, we did not rely solely on *p*-values to judge practical significance (63). To check the chain mediation effect of job satisfaction and burnout between salaries and turnover intentions for the full sample, the model was constructed using the command *gsem* in STATA software version MP17.0, and Bootstrap (10,000) was used to check the chain mediation effect. When the 95% confidence interval (CI) after bias correction does not include zero, it indicates that the pathway has a significant effect. Salary, job satisfaction, burnout, and turnover intention were utilized to construct a chain-mediated effects model, with gender, age, education, overtime, and chronic diseases included as control variables. Chain mediation models for both the married and unmarried subgroups were analyzed and tested following the main model. It should be noted that we employed a chained mediation model to estimate the indirect effect of salary on turnover intention via job satisfaction and job burnout (statistically defined as the product of path coefficients). Given that this study employs a cross-sectional design, the term “mediation” is used here solely in a statistical sense (i.e., to test for indirect effects) and does not imply causal inference. The temporal relationships among the variables are based on theoretical assumptions and have not been empirically measured. In addition, All coefficients reported below are regression-based associations; the terms “direct effect,” “indirect effect,” and “total effect” are used as conventional labels in path analysis and do not imply causality. Salary was log-transformed, and all models were adjusted for covariates, including age, education, job title, and overtime. Using SPSS software version 26.0, reliability was analyzed using Cronbach's alpha coefficients and validity tests of the scales used the KMO values, and assessed its structural validity through the Bartlett test of sphericity and CFA. The rest of the statistical analyses were completed using STATA software version MP17.0, and statistical significance was defined as a two-tailed *p*-value of less than 0.05. Currency conversion was based on the average exchange rate for 2024: 1 USD ≈ 7.1217 CNY.

## 3 Results

The study sample comprised 4,257 females (67.11%); the average age was 39.28; and 5,054 (79.68%) were married. There were 5,543 cases (87.39%) with the *bianzhi;* those with a bachelor's degree accounted for 69.16% of the sample; and primary titles accounted for 37.35%, intermediate titles for 25.11%, and senior titles for 18.51% of the sample. There were 1,240 cases (19.55%) of overtime work in the last six months; and 5.09% worked at the provincial level, 19.45% at prefecture, and 75.45% at the county level CDCs.

### 3.1 Descriptive statistics

From Table 1, the average monthly salary (USD863.23) exhibits significant differences across various sociodemographic subgroups, excluding overtime. The mean scores of job satisfaction (3.52 ± 0.52) and turnover intention (2.36 ± 0.69) varied significantly across sociodemographic subgroups. Except for gender and *bianzhi*, all sociodemographic characteristics also showed significant differences in the burnout score (6.44 ± 2.36). (All *P* < 0.05).

**Table 1:** Salary, job satisfaction, burnout, and turnover intention scores for different characteristics.

Characteristics		Salary (USD), M ±SD	Job satisfaction, M ±SD	Burnout, M ±SD	Turnover intention, M ±SD
Gender	Female	846.91 ± 332.61^**^	3.54 ± 0.50^**^	6.40 ± 2.36	2.32 ± 0.67^***^
	Male	896.54 ± 311.63	3.48 ± 0.55	6.51 ± 2.35	2.45 ± 0.73
Age group	< =25	580.65 ± 378.89^***^	3.67 ± 0.56^***^	6.33 ± 2.56^***^	2.61 ± 0.74^***^
	25 < x < = 40	789.77 ± 312.49	3.48 ± 0.53	6.78 ± 2.42	2.51 ± 0.72
	40 < x < = 55	960.29 ± 283.38	3.54 ± 0.49	6.10 ± 2.23	2.19 ± 0.62
	>=56	1144.49 ± 334.62	3.63 ± 0.45	5.71 ± 1.91	2.06 ± 0.52
*Bianzhi*	Yes	914.60 ± 257.07^***^	3.50 ± 0.51^***^	6.50 ± 2.35	2.34 ± 0.69^***^
	No	507.33 ± 493.46	3.68 ± 0.53	6.03 ± 2.37	2.53 ± 0.69
Degree	Master or above	1142.62 ± 401.05^***^	3.39 ± 0.53^***^	6.98 ± 2.53^***^	2.35 ± 0.70^***^
	Bachelor	858.97 ± 285.12	3.49 ± 0.52	6.59 ± 2.38	2.41 ± 0.72
	Below bachelor	820.39 ± 383.93	3.64 ± 0.50	5.93 ± 2.16	2.23 ± 0.60
Title	Senior	1115.85 ± 276.19^***^	3.53 ± 0.49^***^	6.12 ± 2.18^***^	2.14 ± 0.62^***^
	Intermediate	924.98 ± 240.00	3.46 ± 0.51	6.51 ± 2.31	2.33 ± 0.67
	Primary	790.56 ± 293.64	3.52 ± 0.52	6.60 ± 2.39	2.45 ± 0.72
	No	678.65 ± 360.55	3.61 ± 0.54	6.33 ± 2.47	2.45 ± 0.69
Overwork	Yes	902.52 ± 311.05	3.36 ± 0.56^***^	7.09 ± 2.51^***^	2.48 ± 0.74^***^
	No	853.68 ± 329.67	3.56 ± 0.50	6.28 ± 2.29	2.34 ± 0.68
Group of CDN	0	826.09 ± 336.68^***^	3.57 ± 0.51^***^	6.32 ± 2.31^***^	2.38 ± 0.70^***^
	1	939.36 ± 289.04	3.45 ± 0.52	6.60 ± 2.40	2.32 ± 0.67
	>=2	975.41 ± 269.22	3.35 ± 0.56	6.97 ± 2.47	2.32 ± 0.70
Marital status	Married	897.19 ± 312.61^***^	3.53 ± 0.51^*^	6.31 ± 2.26^***^	2.31 ± 0.66^***^
	Unmarried	730.07 ± 346.07	3.51 ± 0.56	6.94 ± 2.64	2.59 ± 0.76
CDC level	Provincial	1,194.43 ± 477.63^***^	3.46 ± 0.51^*^	6.66 ± 2.54^*^	2.23 ± 0.62^***^
	Prefectural	969.56 ± 329.92	3.51 ± 0.51	6.31 ± 2.40	2.26 ± 0.70
	County	813.46 ± 291.95	3.53 ± 0.52	6.46 ± 2.33	2.40 ± 0.69

^*^P < 0.05, ^**^P < 0.01, ^***^P < 0.001; *P*-values are based on the Chi-square test and Kruskal-Wallis H test. M, mean; SD, standard deviation; USD, United States Dollar; CDN, chronic diseases number.

### 3.2 Common method bias

Since all variables were self-reported, Harman's one-way statistical control method (61, 62) was used to test for common method bias. The percentage of the variance explained by the first factor in the total variance was used as the basis for judgment, with the 26.84% variance not exceeding the critical criterion of 40%, indicating that there was no obvious common method bias.

### 3.3 Association of main variables

Table 2 shows the results of the association analysis between the main variables. Salary was positively associated with job satisfaction, and negatively associated with burnout and turnover intention. Job satisfaction was negatively associated with burnout and turnover intention, whereas burnout was positively associated with turnover intention. All associations were statistically significant (*P* < 0.05), although the associations between salary and job satisfaction, and between salary and burnout, were very weak in magnitude.

**Table 2:** Associations among main variables.

	Salary	Job satisfaction	Burnout	Turnover intention
Salary	1			
Job satisfaction	0.028^*^	1		
Burnout	−0.037^**^	−0.554^***^	1	
Turnover intention	−0.173^***^	−0.431^***^	0.472^***^	1

^*^P < 0.05, ^**^P < 0.01, ^***^P < 0.001.

### 3.4 The chain mediation model for the total sample

Table 3 displays the chain mediation model results for the total sample. After fixing the confounding variables, Table 3 shows that for every 1% increase in respondents' salary, the score of job satisfaction would increase by 0.00165 points (β = 0.165, *P* < 0.001), and the score of turnover intention would decrease by 0.00059 points (β = −0.059, *P* < 0.05). For every 1-point increase in respondents' job satisfaction, the score of burnout would decrease by 2.396 points (β = −2.396, *P* < 0.001) and turnover intention would decrease by 0.349 points (β = −0.349, *P* < 0.001). For every 1-point increase in burnout, the score of turnover intention would increase by 0.088 points (β = 0.088, *P* < 0.001). These results support Hypothesis 1.

**Table 3:** Regression results for the chain mediation model of the total sample.

		Job satisfaction			Burnout			Turnover intention		
		β	R.SE	95% CI	β	R.SE	95% CI	β	R.SE	95% CI
Salary		0.165^***^	0.026	0.113~0.217	0.126	0.091	−0.053~0.304	−0.059^*^	0.026	−0.110~-0.008
Job satisfaction					−2.396^***^	0.055	−2.504~-2.287	−0.349^***^	0.019	−0.386~-0.312
Burnout								0.088^***^	0.004	0.080~0.096
*Control variables*										
Gender(ref: male)	Female	0.063^***^	0.014	0.035~0.091	−0.031	0.054	−0.136~0.074	−0.166^***^	0.016	−0.197~-0.135
Age		0.006^***^	0.001	0.004~0.007	−0.041^***^	0.004	−0.048~-0.033	−0.012^***^	0.001	−0.014~-0.010
*Bianzhi* (ref: no)	Yes	−0.201^***^	0.028	−0.257~-0.145	0.183	0.102	−0.017~0.383	−0.180^***^	0.030	−0.238~-0.121
Degree (ref: below bachelor)	Master or above	−0.162^***^	0.039	−0.238~-0.086	0.125	0.150	−0.168~0.419	0.049	0.041	−0.032~0.130
	Bachelor	−0.089^***^	0.017	−0.123~-0.054	−0.043	0.067	−0.175~0.089	0.034	0.019	−0.002~0.071
Title (ref: no)	Senior	−0.103^***^	0.026	−0.154~-0.051	−0.023	0.102	−0.224~0.178	−0.024	0.029	−0.080~0.033
	Intermediate	−0.140^***^	0.022	−0.184~-0.096	0.008	0.087	−0.163~0.179	0.021	0.024	−0.027~0.069
	Primary	−0.058^**^	0.020	−0.096~-0.019	0.053	0.075	−0.094~0.200	0.026	0.021	−0.015~0.068
Overtime (ref: no)	Yes	−0.162^***^	0.017	−0.195~-0.129	0.293^***^	0.064	0.168~0.419	0.012	0.019	−0.026~0.049
Number of chronic diseases		−0.107^***^	0.010	−0.126~-0.089	0.196^***^	0.037	0.123~0.268	−0.017	0.011	−0.037~0.004
CDC level (ref: county)	Provincial	−0.049	0.033	−0.112~0.015	−0.119	0.131	−0.377~0.138	−0.209^***^	0.037	−0.281~-0.137
	Prefectural	−0.006	0.016	−0.038~0.026	−0.227^***^	0.065	−0.354~-0.100	−0.120^***^	0.019	−0.158~-0.082
_cons		2.238^***^	0.208	1.831~2.645	15.160^***^	0.729	13.732~16.588	4.291^***^	0.217	3.866~4.716

^*^P < 0.05, ^**^P < 0.01, ^***^P < 0.001. β, Regression coefficient; R.SE, Robust standard error; CV, Control variable; 95% CI, the lower and upper 95% confidence intervals; ref, reference level. The baseline level was male workers in county-level CDCs with no bianz*hi*, below bachelor's degree, no title, and no overtime.

Table 4 provides the total, direct, and indirect effects of the model paths. The significance of all effects was checked using 95% bootstrap confidence interval estimates. Except for the indirect effect path 2, all other paths were significant, with the direct path accounting for 42.14% and the indirect effects accounting for 57.86% of the total effect. The model showed that respondents' salary directly affected turnover intention [direct effect, β = −0.059, 95% CI (−0.111, −0.007)]. Job satisfaction played a separate mediating role between salary and turnover intention [Path 1, β = −0.058, 95% CI (−0.076, −0.039)]. This confirms Hypothesis 2. The mediating effect of burnout on the relationship between salary and turnover intention was not significant [95% CI (−0.005, 0.027)], which rejects Hypothesis 3. Job satisfaction and burnout were found to play a chain mediating role between salary and turnover intention [Path 3, β = −0.035, 95% CI (−0.046, −0.023)], which supports Hypothesis 4.

**Table 4:** Total, direct, and indirect effects of model paths for the total sample.

Model paths	Observed β	Bootstrap SE	95% CI (BC)
Total effect	−0.140	0.033	−0.206~-0.075
Direct effect	−0.059	0.026	−0.111~-0.007
Indirect effect	−0.081	0.018	−0.116~-0.047
Path1	−0.058	0.010	−0.076~-0.039
Path2	0.011	0.008	−0.005~0.027
Path3	−0.035	0.006	−0.046~-0.023

Path1, Salary → Job satisfaction → Turnover intention; Path2, Salary → Burnout → Turnover intention; Path3, Salary → Job satisfaction → Burnout → Turnover intention. β, Regression coefficient; SE, Standard error; CI, Confidence intervals; BC, Bias-corrected confidence interval.

### 3.5 The chain mediation model of marriage subgroups

Using the same method and control variables as the total sample, the chained mediated effects model was separately analyzed for the married (Model-1) and unmarried (Model-2) subgroup samples. The main results of the model are displayed in Table 5. After fixing the confounding variables, the results of the model for married respondents showed that for every 1% increase in the respondent's salary, the score of job satisfaction would increase by 0.00214 points (β = 0.214, *P* < 0.001), and the score of turnover intention decreased by 0.00065 points (β = −0.065, *P* < 0.05). For every 1-point increase in respondents' job satisfaction, the score of burnout decreased by 2.230 points (β = −2.230, *P* < 0.001), and turnover intention decreased by 0.302 points (β = −0.302, *P* < 0.001). For every 1-point increase in burnout, the score of turnover intention increased by 0.085 points (β = 0.085, *P* < 0.001). The results of the model for unmarried respondents showed that for every 1-point increase in job satisfaction, the score of burnout decreased by 2.852 points (β = −2.852, *P* < 0.05) and turnover intention decreased by 0.508 points (β = −0.508, *P* < 0.001). For every 1-point increase in burnout, the score of turnover intention increased by 0.090 points (β = 0.090, *P* < 0.001). None of the model coefficients for the relationships between income and job satisfaction, burnout, and turnover intention reached statistical significance (all *P* > 0.05).

**Table 5:** Regression results for the chain mediation model among marital-status subgroups.

	Job satisfaction			Burnout			Turnover intention		
	β.CV	R.SE	95% CI	β.CV	R.SE	95% CI	β.CV	R.SE	95% CI
Model−1: Married									
Salary	0.214^***^	0.031	0.152~0.275	0.109	0.111	−0.110~0.327	−0.065^*^	0.031	−0.126~-0.004
Job satisfaction				−2.230^***^	0.061	−2.349~-2.111	−0.302^***^	0.021	−0.344~-0.261
Burnout							0.085^***^	0.004	0.076~0.094
Model−2: Unmarried									
Salary	0.091	0.048	−0.004~0.185	0.051	0.161	−0.265~0.368	−0.067	0.048	−0.161~0.026
Job satisfaction				−2.852^*^	0.128	−3.103~-2.601	−0.508^***^	0.041	−0.588~-0.427
Burnout							0.090^***^	0.009	0.073~0.107

^*^P < 0.05, ^**^P < 0.01, ^***^P < 0.001. β.CV, Regression coefficient with control variables; R.SE, Robust standard error; 95% CI, the lower and upper 95% confidence intervals. Control variables and reference levels are consistent with the total sample model in Table 3. The baseline level was male workers in county-level CDCs with no bianzhi, below bachelor's degree, no title, and no overtime.

Table 6 provides the total, direct, and indirect effects of the paths for the married (Model-1) and unmarried (Model-2) subgroup samples. The significance of all effects was checked using 95% bootstrap confidence interval estimates. Path results for the married subgroup showed that only Path 1 and Path 3, which represent indirect effects, were significant, accounting for 59.63% of the total effect. The direct path effect of salary on turnover intention among married respondents, as well as the mediating effect of burnout on the relationship between salary and turnover intention (Path 2), were both insignificant (95% CI included 0). Job satisfaction played an independent mediating role between salary and turnover intention [Path 1, β = −0.065, 95% CI (−0.088, −0.045)]. Job satisfaction and burnout were also found to play a chain mediating role between salary and turnover intention [Path 3, β = −0.041, 95% CI (−0.054, −0.029)]. The results of the model analysis for the unmarried sample indicated that job satisfaction (β = −0.508) and burnout (β = 0.090) both had a direct effect on turnover intention; however, neither the direct [95% CI (−0.162, 0.034)] nor the indirect [95% CI (−0.161, 0.005)] effects of salary on turnover intention were significant, and Hypotheses 1–4 are all rejected in the unmarried subgroup.

**Table 6:** Total, direct, and indirect effects of model paths for marital-status subgroups.

Model paths	Model-1: Married			Model-2: Unmarried		
	Observed β	Bootstrap SE	95% CI (BC)	Observed β	Bootstrap SE	95% CI (BC)
Total effect	−0.161	0.038	−0.235~-0.086	−0.132	0.067	−0.263~0.009
Direct effect	−0.065	0.032	−0.127~0.003	−0.067	0.048	−0.162~0.034
Indirect effect	−0.096	0.019	−0.132~-0.057	−0.065	0.041	−0.161~0.005
Path1	−0.065	0.011	−0.088~-0.045	−0.046	0.026	−0.108~0.002
Path2	0.009	0.009	−0.009~0.028	0.005	0.015	−0.026~0.033
Path3	−0.041	0.006	−0.054~-0.029	−0.023	0.012	−0.050~0.001

Path1, Salary → Job satisfaction → Turnover intention; Path2, Salary → Burnout → Turnover intention; Path3, Salary → Job satisfaction → Burnout → Turnover intention. β, Regression coefficient; SE, Standard error; CI, Confidence intervals; BC, Bias-corrected confidence interval.

## 4 Discussion

Our contribution was to investigate the complex inter-relationships between salary, job satisfaction, burnout, and turnover intention. We found a chain mediating effect of job satisfaction and burnout between salaries and turnover intentions among CDC staff in the study province. Previous studies have investigated single variables or only bilateral relationships between salary, job satisfaction, burnout, and turnover intention. For example, a study of local CDCs in Hainan Province reported a job satisfaction level of 3.35 out of 5 points, indicating a moderate degree of satisfaction (35). A survey of Sichuan Province prefectural and county-level CDC staff found that 80.73% experienced burnout (64). An investigation by CDC staff in Henan Province found that the overall job satisfaction score among was low (67.13/100), while their intention to leave was relatively high (7.31/10) (65). A survey of newly recruited CDC personnel in Lianyungang City, Jiangsu Province, revealed a moderate turnover intention level (57.20/100) (66). These findings are broadly consistent with our results, where the level of job satisfaction was moderate (3.52/5), the burnout rate was relatively high (80.83%), and turnover intention was at a moderate level (2.36/5). Our research revealed the independent mediating effect of job satisfaction between the direct effect of salary on turnover intention, and validated the chain mediating effect of job satisfaction and burnout between salary and turnover intention. Our study also found differences in the mediation path for the marital status subgroups suggest heterogeneity within the subsample.

Analysis of the overall sample revealed that salary is directly associated with turnover intentions among CDC staff, with a direct effect of 42.14%, similar to reported results for Australian mental health workers, nurses in Philippine hospitals, and Indian dental hygienists (15–17). The issue of low salary among CDC personnel has been extensively documented (11, 32), with salary the primary consideration for CDC staff in turnover decisions (7–9). For example, a Beijing study found that 54.51% of CDC staff expressed salary dissatisfaction, and 44.48% had considered resigning, with low salary being the key driver (21). Inadequate salaries play a key role in weakening the economic stickiness of job retention and reducing employees' sense of identity and trust in the organization (11). Our recommendation for higher salaries does not imply indiscriminately pursuing higher salaries as a stand-alone approach, but as part of a wider strategy optimizing compensation structures, aligning career development with performance incentives, addressing burnout and job satisfaction, and targeting an optimal salary incentive range. In 2023, the Chinese government proposed reforming the compensation scheme for CDC personnel as a measure to promote the high-quality development of the CDC system (67). Given our mediation results, we suggest that a stand-alone compensation policy is unlikely to be successful.

Our results show that both job satisfaction and burnout are associated with turnover intentions among CDC staff, and job satisfaction is negatively associated with burnout. While these direct relationships have been noted in previous studies (49, 68), our mediated model's full-sample results support two hypotheses. Job satisfaction and burnout exhibit a chained mediating effect between salary and turnover intentions among CDC workers, and job satisfaction demonstrates an independent mediating effect. The cumulative proportion of indirect effects reached 57.86%, with lower salaries leading to lower job satisfaction, triggering this mediation process. When excessive workloads and work-related stress accumulate without a corresponding increase in compensation, such as no significant salary difference between overtime and non-overtime subgroups, CDC staff may feel that their hard work is not fully recognized and that their contributions are undervalued, leading to reduced job satisfaction (7, 69). This is followed by a significant decline in work engagement, when emotionally exhausted staff subconsciously reduce extra effort, performing only the bare minimum required to avoid the feeling of being “exploited,” fostering and intensifying burnout. When this psychological burnout persists without salary increases or other recognitions, CDC staff seek external alternative opportunities, viewing resignation as the ultimate path to restore psychological equity and gain value recognition. Ultimately, this chain reaction—from “perceived mismatch” to “psychological imbalance” and then to “behavioral withdrawal” —led to resignations.

Other studies have indicated that factors such as limited career advancement opportunities, inadequate benefits, and suboptimal working conditions contribute to turnover among CDC staff (7, 69). But improvements in these factors are likely to have only a limited impact. We found that salary stood at the head of the chain effect, which requires CDC administrators to optimize performance incentives and financial management mechanisms. Meanwhile, job satisfaction plays a more central role in the chained mediating effect, highlighting the need to prioritize its improvement. As discussed, salary adjustments alone are unlikely to solve CDC's personnel retention problem without addressing employees' multifaceted developmental needs, job dissatisfaction, burnout, and job satisfaction.

Significantly, our subgroup marital status analysis revealed heterogeneous pathways linking salary, job satisfaction, burnout, and turnover intention. In the unmarried group, no significant associations were observed between salary and job satisfaction, burnout, or turnover intention. This indicates that the economic factors were less significant in the unmarried subgroup. We posit that this inertia operates bidirectionally (24, 70). The family-work spillover theory posits that work-related experiences—including perceptions of whether compensation is adequate—may be influenced by family life and may also influence family life (71). Married individuals are compelled to remain employed due to family financial obligations, but their salary level failed to balance household expenditures and their perceived labor value, triggering a negative feedback cycle between the work and family domains, which accelerated turnover. Further, job dissatisfaction induced by low salaries heightened the likelihood of employee turnover. As the economic dependency theory emphasizes, economic dependence within marriage acts as a stabilizer for employment; financial responsibilities and economic dependence prevent married individuals from quitting their jobs on a whim, while salary levels that fail to meet household expenses and personal sense of worth will intensify the desire to leave (72, 73). While our data confirmed prior studies (14) that lower turnover intentions among married staff (2.31 ± 0.66) vs. unmarried (2.59 ± 0.76) (*P* < 0.01), our explanation of the process is more nuanced. While married workers' heightened economic dependence drove more stable married CDC employment, our marital subgroup analysis provides empirical substantiation for this heterogeneity based on the complex interplay between salaries, perceived work worth, job satisfaction, family-work domain, and turnover. These results reinforce our recommendation to link CDC salary rises with a range of work and family arrangements. For example, improvements in compensation combined with family-friendly policies (such as housing subsidies, child care support, and flexible work arrangements) to address work-family spillover effects.

Although unmarried individuals did not exhibit economic retention inertia, the burnout score among unmarried CDC staff (6.94 ± 2.64) was significantly higher than that of married staff (6.31 ± 2.26) (*P* < 0.01). Existing studies indicate that unmarried—typically younger—workers demonstrate lower marginal utility sensitivity to salary (74). Previous studies on the job preferences of CDC staff have shown that professional development opportunities, not salary, were prioritized by most unmarried workers in their early career stages (47, 75). Therefore, we recommend the design and clear communication of well-defined career development paths, providing growth-oriented incentives and supportive training to enhance young CDC staff's confidence in their professional prospects.

Like Chinese doctors and nurses (18, 23), our analysis showed that work and sociodemographic characteristics, such as gender, age, job title, educational background, overtime work, number of chronic diseases, and unit level, impacted job satisfaction and burnout of CDC staff and directly or indirectly influenced their turnover intentions. To tackle turnover intention, CDC administrators must address the psychological wellbeing of CDC staff (5), particularly among highly educated individuals holding master's degrees or higher and young workers frequently engaged in night shifts and overtime commitments. When refining retention strategies for CDC personnel, policymakers must shape retention policies for the heterogeneous characteristics of employee subgroups. Besides income-related policies, such as reasonable overtime compensation, we recommend that CDC policymakers provide preferential access to research resources for highly educated research-oriented personnel; develop burnout intervention programs for “at-risk” workers; and conduct regular mental health screenings for staff.

We collected a rich research dataset on salaries, job satisfaction, burnout, and turnover intentions across a study province. However, when generalizing these findings, one must take into account specific contexts and varying real-world conditions. Our cross-sectional survey data preclude definitive causal interpretations among key variables. Variables were measured via self-administered questionnaires and self-rated scales. Despite high reliability and validity and rigorous quality control, inherent subjectivity and measurement errors are unavoidable. Future studies should collect further decision-making variables, such as professional identity, social support, and familial stressors. It should also be noted that, while employees are naturally clustered within CDC institutions, we were unable to adjust for this nesting because individual CDC identifiers were not permitted under the data use agreement. We controlled for CDC level (provincial, prefectural, county) to partially account for between-CDC differences, but residual clustering may still affect standard errors. Future studies should collect institutional identifiers to enable multilevel or cluster-robust analyses. In addition, marital status is a key categorical variable, and the higher proportion of married respondents may have influenced some of the results. However, the distributional bias of marital status precisely reflects the actual demographic structure of the CDC workforce. With regard to the core findings of this study, this does not introduce directional or significant bias into the results and has a very limited effect on the reliability of the conclusions. Of course, future studies that include a larger proportion of unmarried or divorced individuals in their samples would help further validate the cross-group stability of this model. Finally, future research should implement longitudinal tracking and cover more provinces and regions to enable more robust causal inference.

## 5 Conclusions

Based on cross-sectional data from all CDCs in a province of China, our study revealed that job satisfaction and burnout demonstrated a chained mediating effect between salary and turnover intention, with job satisfaction additionally exhibiting an independent mediating effect. No comparable mediating role was observed for burnout. The unmarried subgroup was found to lack the turnover stickiness observed in the married subgroup and exhibited a higher burnout score. Given our mediation results, a stand-alone compensation policy is unlikely to be successful in addressing CDC turnover. We recommend a multifaceted marital-specific approach to CDC personnel management that optimizes compensation structures, aligns career development with performance incentives, addresses burnout, and improves job satisfaction.

## Data Availability

The raw data supporting the conclusions of this article will be made available by the authors, without undue reservation.
